# Breaking the Cycle: A Case of Delayed Diagnosis and Sustained Remission Following Definitive Surgical Treatment of Premenstrual Dysphoric Disorder (PMDD)

**DOI:** 10.1155/crps/8233699

**Published:** 2026-09-07

**Authors:** Daniel J. Deaton, Susannah L. Deaton

**Affiliations:** ^1^ Department of Family Medicine, Meritus Family Medicine Residency Program, Hagerstown, Maryland, USA; ^2^ Department of Psychology, Capella University, Minneapolis, Minnesota, USA, capella.edu

**Keywords:** delayed diagnosis, neuroendocrine sensitivity, oophorectomy, physician bias, premenstrual dysphoric disorder (PMDD), reproductive psychiatry, women’s mental health

## Abstract

Premenstrual dysphoric disorder (PMDD) is a hormone‐sensitive mood disorder characterized by cyclical psychiatric and physical symptoms that impair functioning. Despite inclusion in DSM‐5 and ICD‐11, PMDD is frequently misdiagnosed or overlooked due to clinical unfamiliarity, lack of structured assessment, and systemic minimization of hormone‐linked distress. Women routinely face delays in diagnosis and often only receive definitive treatment through self‐advocacy. This report describes a 35‐year‐old cisgender female with treatment‐resistant PMDD, whose symptoms began after menarche and worsened over two decades. Despite multiple psychiatric and medical encounters, her condition was misattributed to obesity, mood disorders, or trauma. After a diagnostic delay and multiple first and second‐line treatment failures, a trial of leuprolide acetate confirmed a hormonal etiology of her symptoms, and she subsequently underwent total hysterectomy with bilateral salpingo‐oophorectomy. Although she experienced a severe depressive episode in the immediate postoperative period, she has since remained in sustained symptom remission on an SSRI and estrogen replacement. This case demonstrates the need to recognize cyclic symptom patterns and potential hormonal etiology in treatment‐resistant mood disorders.

## 1. Introduction

Premenstrual dysphoric disorder (PMDD) is a hormone‐sensitive mood disorder affecting 3%–8% of menstruating individuals [1, 2]. With intense mood lability and varied, severe psychiatric and physical symptoms, PMDD is detrimental to daily functioning and quality of life [2]. Pathophysiology of PMDD is unique among both hormonal and psychiatric disorders, caused not by abnormal hormone levels but by an abnormal response to fluctuations in ovarian hormones that impact GABAergic neuronal signaling [2]. This neuroendocrine sensitivity disrupts emotional regulation, cognitive functioning, and stress reactivity [2]. Despite a lack of measurable biomarkers, tools such as the Daily Record of Severity of Problems (DRSP) and the Premenstrual Symptoms Screening Tool (PSST) are validated assessments to provide an accurate diagnosis [3]. However, these English‐language measures have limited cross‐cultural validation, and there are other DSM‐5‐aligned instruments being validated in other languages and cultural settings [4]. PMDD continues to be misunderstood, misdiagnosed, or inadequately treated across healthcare settings despite its inclusion in the DSM‐5 in 2013 [5, 6].

Patients with PMDD will often describe years of misdiagnosis, dismissal by medical providers, and reliance on self‐advocacy for diagnosis and treatment [5]. Without guidance and structured assessment, many undergo years of ineffective medication trials while facing cyclic psychiatric instability, resulting in prolonged functional impairment, psychiatric hospitalization, and suicide [5–7]. In general, physicians lack familiarity with PMDD’s diagnostic criteria and may mistake it for recurrent major depressive episodes, generalized anxiety, or bipolar disorder [6].

These clinical lapses are propagated by systemic biases that shape how women’s suffering is perceived. Investigation into women’s experiences seeking care have found that patients reporting menstrual‐related psychiatric distress are often dismissed or told their experiences are overly emotional or dramatic [8]. This gendered dismissal is a well‐documented phenomenon in women’s health care, where subjective reports are more frequently discounted and research specific to female physiology is underfunded [9]. This case report presents a woman with treatment‐resistant PMDD whose decades‐long journey toward diagnosis and definitive surgical treatment reflect these systemic barriers.

## 2. Case Presentation

The patient is a 35‐year‐old cisgender female with a history of mood instability, chronic depression, obsessive–compulsive disorder, polycystic ovary syndrome (PCOS), and obesity, later diagnosed with PMDD. Her severe, cyclic symptoms began following menarche, with episodes meeting PMDD DSM‐5 diagnostic criteria for both psychiatric and physical symptoms that worsened over decades, according to retrospective symptom review. Despite numerous encounters with the healthcare system, her symptoms were attributed to PCOS, generalized anxiety, major depressive disorder, or trauma‐related stress. Multiple medication trials and hormonal interventions failed to provide relief, and a diagnosis with effective treatment was not achieved until her mid‐30s.

The patient’s early development was marked by untreated ADHD, anxiety, depressive symptoms, and obsessive–compulsive traits, along with significant childhood adversity including familial abuse and nonfamilial sexual trauma. Menarche occurred at age 12, after which she began experiencing pronounced cyclical mood symptoms of depression, irritability, intrusive thoughts, bloating, and affective lability that worsened in the luteal phase. These symptoms were attributed to generalized anxiety or major depression by her primary care physician. Multiple SSRI trials were ineffective or poorly tolerated in adolescence, including fluoxetine 60 mg daily, which produced chest pain as a side effect. COCs (combined oral contraceptives) were initiated for menorrhagia and dysmenorrhea but did not improve her psychiatric symptoms.

In early adulthood, the patient experienced worsening monthly mood lability and profound lethargy that led to leaves of absence from the university. These episodes were also marked by anhedonia, musculoskeletal pain, irritability, and a recurrent sense of emotional loss of control. During one episode, she sought psychiatric care but was told that no treatment would be provided at the intake visit. She attempted suicide later that day, and subsequent psychiatric care did not recognize her cyclic symptoms. During her early 20s, she also experienced severe intimate partner violence. At age 26, she was diagnosed with PCOS based on all three Rotterdam criteria and was prescribed various hormonal contraceptives, including COCs, a vaginal ring, and a levonorgestrel‐releasing intrauterine device. Despite trials of at least 3 months each, none improved her mood symptoms. Psychotherapy was pursued intermittently from age 27–29 but was discontinued due to a lack of improvement. Over the course of her 20s, many other antidepressants and contraceptives were attempted without significant benefit. Refer to Table 1 for a full list of pharmacologic interventions.

**Table 1:** Pharmacologic treatment trials by age, indication, dose, duration, and patient‐reported outcome.

Age	Working diagnosis	Treatment	Maximum dose achieved	Duration	Response	Adverse effect	Reason discontinued
Age 13–17	GAD/MDD	Fluoxetine	60 mg daily	—	No improvement	Chest pain	Adverse effect
Age 13–17	GAD/MDD	Additional SSRI trials	—	—	No improvement	—	Ineffective
Age 13–17	Menorrhagia, dysmenorrhea	COC	—	—	No mood improvement	—	Ineffective
Age 18–25	GAD/MDD	Mirtazapine	30 mg nightly	≥3 months	No mood improvement	Excessive somnolence	Adverse effect
Age 26	Menorrhagia, dysmenorrhea	Progestin‐only pill	—	≥3 months	No mood improvement	—	Ineffective
Age 26	Dysmenorrhea	Vaginal ring	—	≥3 months	No mood improvement	—	Ineffective
Age 27–29	PCOS	LNG‐IUD	—	≥12 months	No mood improvement	—	Desiring pregnancy
Age 28	GAD/MDD	Retrial fluoxetine	40 mg daily	≥12 months	Minor mood improvement	—	Incomplete response
Age 28	GAD/MDD	Bupropion	300 mg daily	≥6 months	Minor mood improvement	Jaw clenching, tremor, anxiety	Incomplete response, adverse effect
Age 29	GAD/MDD	Retrial mirtazapine	15 mg nightly	<3 months	No mood improvement	Excessive somnolence	Adverse effect
Age 30	GAD/MDD	Venlafaxine	150 mg daily (Extended release)	≥6 months	Minor mood improvement	Anxiety, hyperhidrosis	Adverse effect
Age 30	GAD/MDD	Duloxetine	30 mg daily	≥3 months	No mood improvement	—	Ineffective
Age 33	PCOS	Drospirenone/ethinyl estradiol	3 mg/0.03 mg	≥3 months	No mood improvement No improvement in menorrhagia	—	Ineffective
Age 33	Bipolar disorder	Aripiprazole	5 mg daily	<3 months	No improvement	Akathisia	Adverse effect
Age 33	Bipolar disorder	Quetiapine	50 mg nightly	<3 months	No improvement	Visual hallucinations	Adverse effect
Age 34	Bipolar disorder	Valproate	500 mg BID	<3 months	No improvement	Weight regain	Ineffective, adverse effect
Age 34	PMDD	Leuprolide acetate	3.75 mg IM monthly	3 months	Marked improvement (self‐reported)	—	Transitioned to surgery
Age 34	PMDD	Estradiol	1 mg oral daily	3 months	Marked improvement (self‐reported)	—	Transitioned to surgery
Postoperative	Postoperative depressive episode	Lamotrigine	—	<2 weeks	Unable to assess due to treatment duration	Mucocutaneous reaction	Adverse effect
Current	PMDD, postbilateral salpingo‐oophorectomy	Escitalopram	5 mg daily	>1 year	Sustained remission	—	Ongoing
Current	PMDD, postbilateral salpingo‐oophorectomy	Estradiol patch	0.1 mg/day	>1 year	Sustained remission	—	Ongoing

*Note:* Dashes indicate unavailable or not applicable data.

In her 30s, the patient experienced substantial symptom relief during two pregnancies but developed severe postpartum symptom exacerbations starting 2–4 weeks after both deliveries. Afterward, her menstrual cycles and premenstrual symptoms became increasingly irregular, and she began consistent prospective monitoring with an unstructured symptom diary. Despite another trial of COCs, her menstrual irregularity did not improve. Throughout her life, she had struggled with obesity and was advised that weight loss would improve both mood and menstrual regularity. Motivated by this, she underwent bariatric surgery at age 33 and achieved a significant, sustained weight loss. Unfortunately, her psychiatric symptoms and menstrual irregularity persisted. As her cycles became less predictable, the irregular timing of mood symptoms contributed to a misdiagnosis of bipolar disorder. She was prescribed antipsychotics, including aripiprazole and quetiapine, which led to akathisia and visual hallucinations. Valproate was later trialed, resulting in weight regain without symptom improvement.

At age 34, after tracking her symptoms and researching PMDD independently, the patient presented published, evidence‐based treatment recommendations for treatment‐resistant PMDD to a provider willing to consider a hormonal etiology for her symptoms. A diagnostic trial of leuprolide acetate, a gonadotropin‐releasing hormone (GnRH) agonist, was initiated along with oral estradiol add‐back. The patient self‐reported marked symptom improvement during the leuprolide acetate trial, which was sustained over several months. She specifically reported a resolution of major, previously limiting symptoms of affective lability, irritability, depressed mood, anhedonia, and lethargy. Given her response to ovarian suppression, extensive history of treatment failure, and the impact her symptoms had on quality of life, she elected to pursue definitive surgical treatment. She underwent a total hysterectomy with bilateral salpingo‐oophorectomy. The psychiatric risks of bilateral oophorectomy were not reviewed with the patient prior to surgery. Approximately 3 weeks postoperatively, when estrogen replacement was being titrated, she experienced a severe depressive episode culminating in a suicide attempt. A brief trial of lamotrigine was initiated during the resulting inpatient psychiatric admission but discontinued due to a mucocutaneous reaction.

At the time of writing, the patient has had sustained self‐reported remission of cyclic symptoms for 1 year following her psychiatric hospitalization. She reports no physical or psychiatric symptom exacerbations and is maintained on estrogen replacement and escitalopram 5 mg daily. Her interpersonal, occupational, and emotional functioning have markedly improved, and she reports for the first time in her adult life a sustained sense of psychological stability.

## 3. Discussion

**Figure 1:**
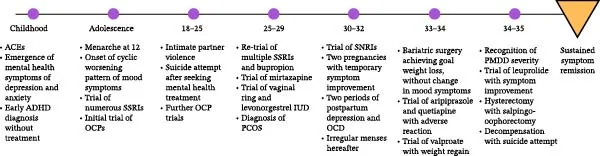
Case timeline from childhood to symptom remission, including medication trials, suicide attempts, diagnosis of PCOS, pregnancies, bariatric surgery, and leuprolide trial followed by hysterectomy with salpingo‐oophorectomy.

This case illustrates the clinical complexity and systemic neglect often faced by individuals with PMDD. Despite experiencing cyclical symptoms since adolescence, the patient remained undiagnosed for over two decades (Figure 1). Her case reflects a well‐documented pattern in which PMDD is underrecognized, misdiagnosed, and misunderstood within psychiatric and primary care settings [5, 6]. The failure to integrate symptom timing into diagnostic evaluations reflects not a one‐time clinical oversight but a broader neglect of women’s health in research and practice [9]. Of unclear significance to the presentation is the patient’s complicated history of adverse childhood events and intimate partner violence. Trauma exposure, especially in childhood, has been associated with premenstrual disorders, though this is not uniform across studies [2]. In this case, however, it obfuscated the clinical picture and contributed to misdiagnosis.

These oversights and diagnostic delays had life‐threatening consequences. Although symptom review was retrospective, the patient’s first suicide attempt appeared temporally linked to the luteal phase of her menstrual cycle, consistent with the expected elevation in suicidal ideation and suicide attempts in unrecognized or undertreated PMDD [7]. Without proper recognition of the cyclical nature of her symptoms, her presentation was repeatedly misattributed to other psychiatric disorders. She was diagnosed with bipolar disorder despite the absence of episodes meeting DSM‐5 criteria for hypomania or mania and no family history of bipolar disorder.

Adding to the physician bias toward this patient was her history of PCOS and obesity. Physicians frequently suggested that weight loss would improve both her mood and menstrual regularity without further addressing her concerns. While weight loss may improve metabolic profiles and cycle regulation in PCOS, in this case, further interventions weren’t pursued while awaiting weight loss [10]. Beyond bias, PCOS contributed to diagnostic confusion through cycle irregularity, obscuring the association of her mood symptoms with the luteal phase of her menstrual cycle. After struggling with weight for years, the patient underwent bariatric surgery, with no change in self‐reported psychiatric symptoms despite a significant and sustained weight loss. This suggests that hormonal sensitivity, rather than obesity, was the primary driver of symptom severity in this case and indicates that bias toward physical phenotypes can lead to dismissing subjective symptoms as exaggerated or not requiring standard interventions beyond weight loss [5, 8].

A further differential consideration in this case is separating PMDD from premenstrual exacerbation (PME) of an underlying psychiatric disorder. PME, while not a DSM‐5 or ICD‐10 diagnosis, is an important contributing factor that can be distinguished from isolated PMDD via periods of symptom‐free intervals during the follicular phase, a distinction that requires close prospective symptom tracking and regular menstrual cycles [2]. In this case, the patient’s mood symptoms demonstrated cyclic periods of remission, with improved quality of life and normal functioning, which was supported by prospective symptom monitoring in her early 30s. However, PME remains a differential consideration and, with the patient’s history of comorbid psychiatric disorders, a likely contributor outside of her primary mood symptoms. The symptom‐free interval during pregnancies, followed by severe postpartum depression and OCD, also supports the role of hormonal sensitivity in her presentation.

**Figure 2:**
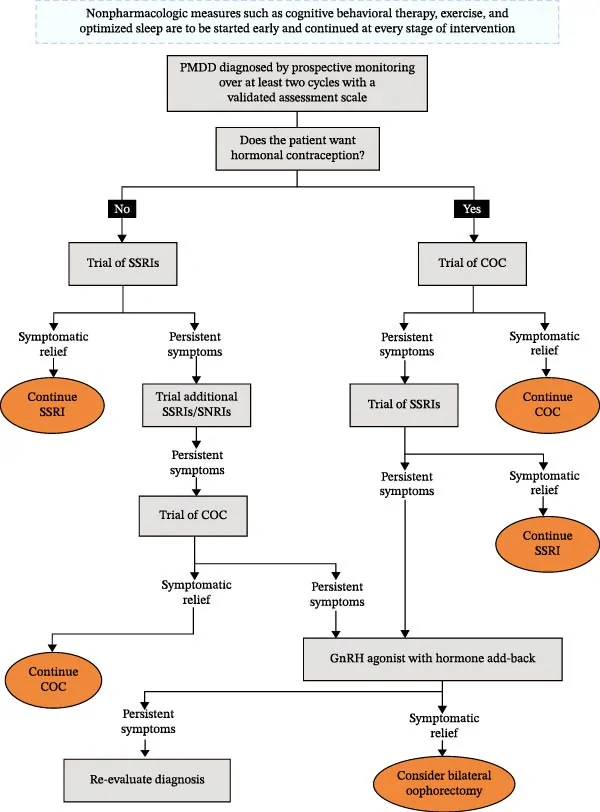
Proposed treatment‐resistant PMDD treatment algorithm, constructed by the authors from published treatment recommendations, reflecting the evidence the patient presented during her clinical encounter prior to GnRH agonist initiation [2, 11, 12].

The first‐line pharmacotherapies for PMDD are SSRIs and COCs [2]. High‐quality evidence suggests that SSRIs likely reduce overall premenstrual symptoms, with ongoing debate on the efficacy of cyclic versus continuous dosing [11]. While COCs appear more effective than placebo for overall premenstrual symptoms, evidence does not consistently support their benefit for premenstrual depressive symptoms. Failing these, hormonal suppression through GnRH agonists is a validated approach in treatment‐resistant PMDD [12]. Immediate side effects are likely to include hot flashes, insomnia, weight changes, body aches, acne, and hair loss. In this case, leuprolide served the dual purposes of providing symptomatic relief while confirming that surgical ovarian suppression would be a valid long‐term treatment option. Figure 2 demonstrates a proposed stepwise approach to treatment‐resistant PMDD as supported by this case, starting from nonpharmacologic measures, and ending with bilateral oophorectomy as an option following GnRH agonist response.

Bilateral oophorectomy has been used as a definitive treatment for severe, treatment‐resistant premenstrual disorders refractory to other interventions, with remission of symptoms reported in over 90% of cases in one uncontrolled cohort [11]. The patient’s severe postoperative depression is a known risk associated with acute estrogen withdrawal, particularly in individuals with a history of prior psychiatric instability [13]. While there is evidence that hysterectomy with and without bilateral oophorectomy is associated with new mental health diagnoses, especially in younger patients, these consequences can be fully or partially mitigated with hormone replacement [14, 15]. Notably, the psychiatric risks of oophorectomy had not been reviewed with the patient preoperatively, and estrogen replacement had not been titrated to the final dose at the time of decompensation. This postoperative outcome emphasizes the risks associated with surgical treatment, as well as the need for close multidisciplinary follow‐up in the immediate postoperative phase.

## 4. Conclusion

This case supports the importance of recognizing cyclical patterns in menstruating patients with treatment‐resistant mood symptoms. This is ideally done with prospective daily symptom monitoring, though it can be strongly suspected with a good retrospective history. Once the diagnosis is confirmed, treatment with SSRIs and COCs remains the first line, with GnRH agonists remaining an option for treatment‐resistant cases. If GnRH agonists provide substantial symptom relief, oophorectomy is an aggressive approach that may be uncomfortable for many physicians to consider but remains an effective, evidence‐based intervention for treatment‐resistant cases. However, such an aggressive treatment option should not be pursued without multidisciplinary preoperative evaluation, extensive counseling on perioperative and postoperative risks, and close multidisciplinary follow‐up. The delays in this patient’s care did not result from a lack of access to care but rather from a lack of recognition of the symptom course leading to repeated misdiagnoses. Misdiagnoses such as these cannot be attributed to knowledge gaps alone but are part of a larger pattern of bias in women’s health care. In this case, the cost of this pattern was decades of ineffective treatments, repeated misdiagnosis, and a suicide attempt before the correct diagnosis was made. After diagnosis and exhaustion of all other reasonable treatment options, this patient achieved sustained symptom remission following definitive surgical treatment.

## Author Contributions

**Daniel J. Deaton**: resources, writing – original draft, supervision. **Susannah L. Deaton**: conceptualization, writing – review and editing, visualization.

## Funding

This research did not receive any specific grant from funding agencies in the public, commercial, or not‐for‐profit sectors.

## Disclosure

The second author is the patient described in this case report and provided written informed consent for publication, including consent for disclosure of this relationship. The first author is her spouse. The first author was not involved in the patient’s clinical care at any point and did not serve in a treating capacity. His role was limited to verification of the factual clinical record and preparation of the manuscript. Both authors acknowledge that this relationship may introduce bias in the interpretation and framing of the case, and have endeavored to present the clinical course accurately and in accordance with the documented medical history.

## Ethics Statement

This case report did not require institutional review board approval, as single‐patient case reports do not meet the regulatory definition of human subjects research at the authors’ institution. The report was prepared in accordance with ICMJE recommendations and CARE 2013 reporting guidelines. Written and informed consent was obtained from the patient for publication of this case report.

## Conflicts of Interest

The authors declare no conflicts of interest.

## Data Availability

Data sharing is not applicable to this article, as no datasets were generated or analyzed during the current study.

## References

[1] Cary E. and Simpson P. , Premenstrual Disorders and PMDD—A Review, Best Practice & Research Clinical Endocrinology & Metabolism. (2024) 38, no. 1, 10.1016/j.beem.2023.101858, 101858. 38182436

[2] Hantsoo L. and Epperson C. N. , Premenstrual Dysphoric Disorder: Epidemiology and Treatment, Current Psychiatry Reports. (2015) 17, no. 11, 10.1007/s11920-015-0628-3, 87. 26377947 PMC4890701

[3] Appleton S. M. , Premenstrual Syndrome: Evidence-Based Evaluation and Treatment, Clinical Obstetrics & Gynecology. (2018) 61, no. 1, 52–61, 10.1097/GRF.0000000000000339. 29298169

[4] Korkmaz Ş.A. , Ertekin H. , Yapar S. , and Aperribai L. , Psychometric Properties of the Turkish Version of the Premenstrual Dysphoric Disorder Questionnaire for DSM-5 (CTDP–DSM–5), BMC Psychology. (2025) 13, no. 1, 10.1186/s40359-025-03576-1, 1277. 41254748 PMC12625172

[5] Chan K. , Rubtsova A. A. , and Clark C. J. , Exploring Diagnosis and Treatment of Premenstrual Dysphoric Disorder in the U.S. Healthcare System: A Qualitative Investigation, BMC Women’s Health. (2023) 23, no. 1, 10.1186/s12905-023-02334-y, 272. 37198676 PMC10193729

[6] Nayak A. , Wood S. N. , and Hantsoo L. , Barriers to Diagnosis and Treatment for Premenstrual Dysphoric Disorder (PMDD): A Scoping Review, Reproductive Sciences. (2025) 32, no. 6, 1757–1767, 10.1007/s43032-025-01861-3. 40251463

[7] Eisenlohr-Moul T. , Divine M. , and Schmalenberger K. , et al.Prevalence of Lifetime Self-Injurious Thoughts and Behaviors in a Global Sample of 599 Patients Reporting Prospectively Confirmed Diagnosis With Premenstrual Dysphoric Disorder, BMC Psychiatry. (2022) 22, no. 1, 10.1186/s12888-022-03851-0, 199. 35303811 PMC8933886

[8] Ng I. K. S. , Tham S. Z. L. , Singh G. D. , Thong C. , and Teo D. B. , Medical Gaslighting: A New Colloquialism, The American Journal of Medicine. (2024) 137, no. 10, 920–922, 10.1016/j.amjmed.2024.06.022. 38936758

[9] Galea L. A. M. and Parekh R. S. , Ending the Neglect of Women’s Health in Research, BMJ. (2023) 381, 10.1136/bmj.p1303, 1303. 37308180

[10] Kataoka J. , Olsson M. , and Lindgren E. , et al.Effects of Weight Loss Intervention on Anxiety, Depression and Quality of Life in Women With Severe Obesity and Polycystic Ovary Syndrome, Scientific Reports. (2024) 14, no. 1, 10.1038/s41598-024-63166-w, 13495. 38866860 PMC11169487

[11] Carlini S. V. , Lanza di Scalea T. , McNally S. T. , Lester J. , and Deligiannidis K. M. , Management of Premenstrual Dysphoric Disorder: A Scoping Review, International Journal of Women’s Health. (2022) 14, 1783–1801, 10.2147/IJWH.S297062. PMC9790166 36575726

[12] Wagner-Schuman M. , Kania A. , Barone J. C. , Ross J. M. , Mulvihill A. , and Eisenlohr-Moul T. A. , What’s Stopping Us? Using GnRH Analogs With Stable Hormone Addback in Treatment-Resistant Premenstrual Dysphoric Disorder: Practical Guidelines and Risk-Benefit Analysis for Long-Term Therapy, The Journal of Clinical Psychiatry. (2023) 84, no. 4, 10.4088/JCP.22r14614, 22r14614. 37341478

[13] Schmidt P. J. , Ben Dor R. , and Martinez P. E. , et al.Effects of Estradiol Withdrawal on Mood in Women With Past Perimenopausal Depression: A Randomized Clinical Trial, JAMA Psychiatry. (2015) 72, no. 7, 714–726, 10.1001/jamapsychiatry.2015.0111. 26018333 PMC6391160

[14] Laughlin-Tommaso S. K. , Satish A. , Khan Z. , Smith C. Y. , Rocca W. A. , and Stewart E. A. , Long-Term Risk of de Novo Mental Health Conditions After Hysterectomy With Ovarian Conservation: A Cohort Study, Menopause. (2020) 27, no. 1, 33–42, 10.1097/GME.0000000000001415. 31479034 PMC7089568

[15] Nitschke A. S. , do Valle H. A. , Dawson L. , Kwon J. S. , and Hanley G. E. , Long-Term Non-Cancer Risks in People With BRCA Mutations Following Risk-Reducing Bilateral Salpingo-Oophorectomy and the Role of Hormone Replacement Therapy: A Review, Cancers. (2023) 15, no. 3, 10.3390/cancers15030711, 711. 36765666 PMC9913268

